# Avoiding False-Positive Glaucoma Diagnosis in Myopic Eyes: Clinical Importance of OCT Scan Diameter

**DOI:** 10.3390/jcm15041669

**Published:** 2026-02-23

**Authors:** Saadet Gültekin Irgat, Ramazan Demirel, Ecem Ulutürk, Alpaslan Koç, Fatih Özcura, Özlem Arık

**Affiliations:** 1 Department of Ophthalmology, Faculty of Medicine, Kutahya Health Sciences University, 43100 Kutahya, Turkey; 2 Department of Biostatistics and Medical Informatics, Faculty of Medicine, Necmettin Erbakan University, 42090 Konya, Turkey

**Keywords:** myopia, glaucoma, retinal nerve fibre layer, spectral-domain OCT, circumpapillary scan diameter, colour-code distribution

## Abstract

**Background/Objectives:** Diagnosing glaucoma in myopic eyes remains challenging because myopia-related structural changes can mimic glaucomatous damage on optical coherence tomography (OCT). This study aimed to identify the optimal circular scan diameter for differentiating physiological myopic thinning from glaucomatous loss by analyzing retinal nerve fibre layer thickness (RNFLT) and colour-code distribution across three scan diameters. **Methods:** In this prospective cross-sectional study, 204 eyes (41 controls, 44 mild myopia, 66 moderate myopia, and 53 high myopia) were examined using spectral-domain OCT (Spectralis, Heidelberg). Three concentric circumpapillary scans centred on the Bruch’s membrane opening were obtained: C1 = 3.5 mm, C2 = 4.1 mm, and C3 = 4.7 mm. Global and sectoral RNFLT were evaluated in seven anatomical regions (TS, NS, N, NI, TI, T, and G). Statistical analyses included one-way ANOVA, repeated-measures ANOVA, and receiver operating characteristic (ROC) analysis. **Results:** RNFLT decreased significantly with increasing scan diameter (*p*< 0.001). Thinning was most pronounced in the nasal-superior (NS) and nasal-inferior (NI) quadrants. Across all diameters, C2 (4.1 mm) showed the highest and most consistent discriminatory performance between myopic and control eyes (NI: AUC = 0.674, *p* = 0.001; NS: AUC = 0.625, *p* = 0.014). A progressive shift in OCT colour-code distribution was observed from green to yellow/red with both increasing myopia and larger scan diameters, reflecting anatomical stretching in the nasal and inferior regions. This change was most prominent at the outer ring (C3), where the temporal-inferior (TI) quadrant showed a significant rise in yellow codes (*p* = 0.020). Repeated-measures ANOVA revealed significant between-group effects in NS and NI (*p* < 0.01) and notable group × diameter interactions in NS and TS (*p*< 0.05). **Conclusions:** RNFLT thinning in non-glaucomatous myopic eyes predominantly affects nasal quadrants, whereas temporal segments remain relatively preserved. The 4.1 mm (C2) scan provides the most balanced diagnostic performance and minimizes false-positive “red disease” results. Recognition of the ring-dependent colour-code shift and segment-specific thinning is crucial for accurate interpretation of OCT findings in myopic eyes.

## 1. Introduction

Myopia is the most prevalent refractive error worldwide, and its incidence is increasing at an alarming rate. Current projections estimate that nearly 5 billion individuals will be affected by 2050 [1]. Beyond its refractive implications, myopia is strongly associated with potentially vision-threatening complications, including myopic macular degeneration, retinal detachment, glaucoma, and cataract formation [2]. Robust epidemiological evidence has established myopia as an independent risk factor for primary open-angle glaucoma (POAG), with the risk approximately doubling in myopic individuals and increasing up to seven-fold in cases of high myopia [3].

Diagnosing glaucoma in myopic eyes remains a considerable clinical challenge. Although retinal nerve fibre layer (RNFL) thinning is a hallmark of glaucomatous damage, similar structural attenuation frequently occurs in myopia as a consequence of axial elongation. Therefore, distinguishing true glaucomatous pathology from physiological myopic alterations is critical. As comprehensively reviewed by Tan et al. [4], this diagnostic dilemma largely stems from myopia-related structural changes, including enlarged or tilted optic discs, axial elongation, and the development of parapapillary gamma and delta zones, all of which may mimic glaucomatous morphology [3,5,6,7].

In the context of precision medicine, ocular imaging has undergone a paradigm shift driven by increasingly sophisticated diagnostic technologies. Wang et al. highlighted this evolution by demonstrating the utility of hyperspectral imaging for the optical identification of diabetic retinopathy, emphasizing the expanding role of high-dimensional data in detecting complex ocular pathologies [8]. Nevertheless, although advanced modalities such as OCT-angiography (OCT-A) and artificial intelligence–based analyses have been proposed to enhance diagnostic accuracy, they present important limitations in myopic populations. Despite being regarded as state-of-the-art, OCT-A diagnostic performance may be compromised by projection artefacts and segmentation errors, particularly in eyes with extreme retinal thinning, as reported by Cakir et al. [6].

Given the uncertainties associated with vascular and functional assessments, structural optical coherence tomography (OCT) remains indispensable for glaucoma screening. However, its reliability is also affected by myopia-related anatomical alterations. Several studies have demonstrated that progressive myopia is associated with significant global RNFL thinning [9,10,11], frequently resulting in the so-called red disease, wherein RNFL values are falsely classified as abnormal [7,12]. A major contributing factor is the limitation of standard imaging protocols; Suwan et al. demonstrated that segmentation errors occur significantly more often in myopic eyes due to peripapillary structural deformation [13]. Although alternative parameters, including Ganglion Cell Complex (GCC) analysis [14] and three-dimensional neuroretinal rim assessment [15], have been proposed, circumpapillary RNFL thickness remains the principal screening parameter in routine clinical practice [12].

At present, the 3.5 mm diameter circular scan centred on the optic nerve head (ONH) constitutes the standard OCT protocol for evaluating circumpapillary RNFL thickness and is incorporated as the default setting in most commercially available devices [16,17]. However, scan diameter significantly influences measurement outcomes, as RNFL thickness physiologically decreases with increasing distance from the optic disc margin [18]. Although comparative studies examining different scan diameters are relatively limited, investigations employing multiple concentric circles—C1 (3.5 mm), C2 (4.1 mm), and C3 (4.7 mm)—have demonstrated that scan diameter selection substantially affects diagnostic performance [19,20]. Smaller circles (C1) may offer greater sensitivity for detecting early glaucomatous damage [21]; however, they are particularly susceptible to confounding effects. As reported by Leung et al. [22], Savini et al. [23], and Chang and Singh [7], the conventional 3.5mm scan trajectory frequently traverses areas of parapapillary atrophy (PPA) and structural deformation commonly observed in myopic eyes, leading to RNFL peak displacement and segmentation artefacts. In contrast, recent studies by Wang et al. [24] and Jiravarnsirikul et al. [3] suggest that larger scan diameters (C2–C3) may provide more specific measurements by sampling retinal regions with relatively preserved anatomy, albeit with a potential reduction in sensitivity due to physiological thinning.

The present study aimed to compare peripapillary RNFL measurements obtained using three different circular scan diameters (C1, C2, and C3) in non-glaucomatous myopic eyes, evaluating both absolute thickness values and colour-coded classification patterns. Our objective was to identify the scan diameter that produces measurements most closely approximating those of healthy controls, thereby improving the differentiation between myopia-related structural alterations and true glaucomatous damage.

## 2. Materials and Methods

### 2.1. Study Design and Participants

This prospective, cross-sectional, single-centre study was conducted in accordance with the tenets of the Declaration of Helsinki and received approval from the Non-Interventional Clinical Research Ethics Committee of the University of Health Sciences (Approval No: 2023/05-21, Date: 25 April 2023).

To ensure the study findings wereapplicable to general practice, the myopic participants were selected from consecutive patients attending routine ophthalmic outpatient examinations at Kütahya Health Sciences University between June 2022 and January 2023, rather than from a pre-screened or referral-based population. This recruitment strategy was specifically adopted to minimize selection bias and reflect real-world clinical practice.

**Figure 1:**
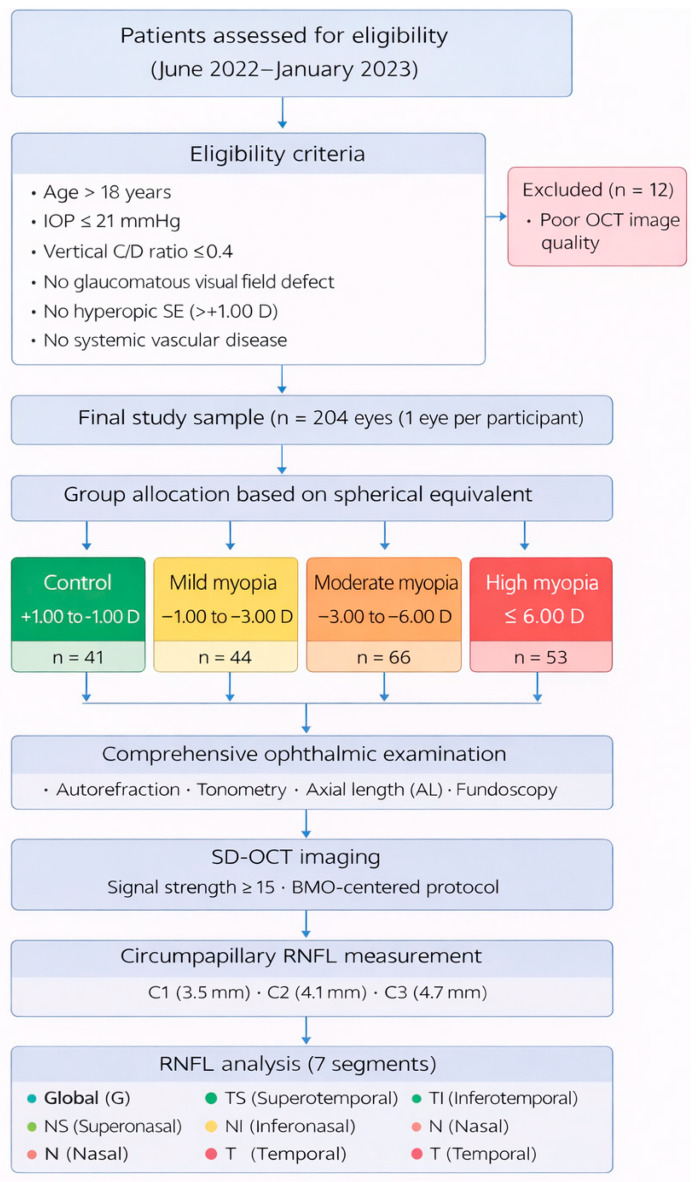
Study flow diagram illustrating participant recruitment, eligibility assessment, stratification according to refractive error, comprehensive ophthalmic examination, SD-OCT imaging protocol (signal strength ≥15 dB; BMO-centred acquisition), and circumpapillary RNFL measurement across three scan diameters (C1: 3.5 mm, C2: 4.1 mm, and C3: 4.7 mm).

Rationale for Inclusion/Exclusion: Eligible participants were aged >18 years. We strictly excluded patients with any signs of glaucomatous optic neuropathy (intraocular pressure [IOP] >21 mmHg, vertical cup-to-disc [C/D] ratio >0.4, or glaucomatous visual field defects) to ensure that RNFL thinning could be attributed solely to myopic stretching rather than pathological damage. Furthermore, participants with systemic conditions (e.g., diabetes mellitus, autoimmune diseases) were excluded to eliminate potential vascular confounders that could alter retinal microcirculation. To focus specifically on axial elongation mechanics, eyes with non-axial myopia or hyperopic spherical equivalent (>+1.00 D) were also removed from the analysis.The participant screening and selection process is summarized in Figure 1.

### 2.2. Ophthalmic Examination and Grouping

All participants underwent a comprehensive ophthalmic evaluation, including autorefractometry and non-contact tonometry (Tonoref III NT-530, Nidek Co., Ltd., Aichi, Japan), best-corrected visual acuity (BCVA) assessment using the Snellen chart, slit-lamp biomicroscopy, dilated fundus examination, and axial length (AL) measurement using the AL-Scan (software version 1.06.01; Nidek Co., Ltd., Gamagori, Japan). Standard automated perimetry was performed using the Humphrey Field Analyzer II-750 (Carl Zeiss Meditec, Dublin, CA, USA). Eyes with confirmed glaucomatous visual field defects were excluded. 

Myopic fundus alterations were classified according to the International Photographic Classification and Grading System for Myopic Maculopathy [25]. Only eyes classified as Category 0 (no myopic degeneration) or Category 1 (tessellated fundus) were included to avoid the confounding segmentation artefacts caused by severe pathologic changes.

Participants were stratified into four groups based on refractive error: control group (+1.00 to −1.00 D), mild myopia (−1.00 to −3.00 D), moderate myopia (−3.00 to −6.00 D), and high myopia (> −6.00 D).

### 2.3. Data Selection Protocol

For statistical analysis, only one eye per participant was included. The selection logic was as follows: when both eyes met the inclusion criteria, the more myopic eye was selected; if refractive error was identical between eyes, the right eye was analyzed. This strategy was applied to ensure statistical independence and to avoid the error of intra-subject correlation, which can artificially inflate the significance of findings in bilateral eye studies.

The study groups demonstrated an acceptable distribution across refractive error categories, without extreme imbalance that could compromise comparative analyses. Only one eye per participant was included to ensure patient-level independence. The dataset encompassed a broad range of myopia severities and axial lengths, introducing structural heterogeneity reflective of routine clinical populations.

### 2.4. SD-OCT Imaging and Scan Protocols

All examinations were performed using the Spectralis SD-OCT system (version 5.4b; Heidelberg Engineering, Heidelberg, Germany). High-resolution radial and peripapillary scans were centred on the Bruch’s membrane opening (BMO) to minimize bias related to optic disc tilt and peripapillary atrophy in myopic eyes [26,27]. Only images with signal strength ≥15 dB were included.

**Figure 2:**
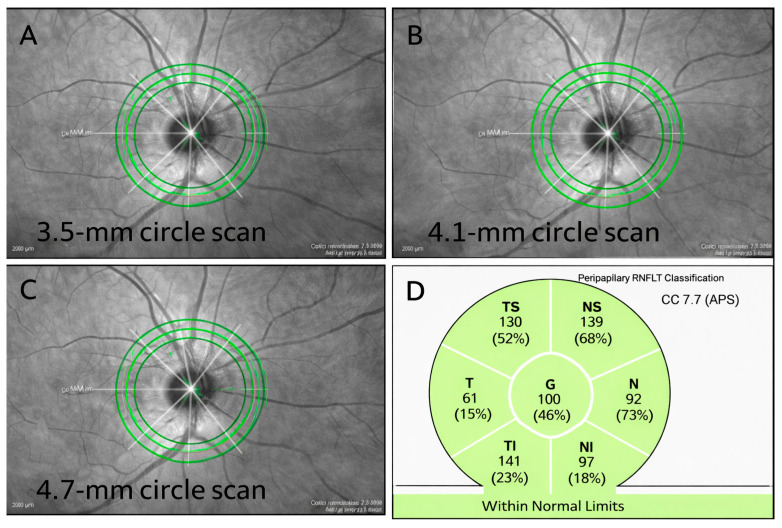
(**A**–**C**) Circumpapillary scan diameters of 3.5, 4.1, and 4.7 mm used for RNFL thickness (RNFLT) measurement in a normal eye; green concentric circles indicate the scan diameters. (**D**) Seven RNFLT parameters, including mean global (G) and sectoral values for the temporal-inferior (TI), inferonasal (NI), superotemporal (TS), superonasal (NS), nasal (N), and temporal (T) sectors, illustrated for the 3.5-mm scan; green areas indicate the normal reference range.

Scan Parameters and Rationale: In addition to the standard 3.5 mm scan (C1), larger diameters of 4.1 mm (C2) and 4.7 mm (C3) were acquired (Figure 2). These wider scans were specifically selected to bypass peripapillary atrophy and minimize segmentation artefacts, a strategy supported by Leung et al. [22] and Wang et al. [24].

Data Analysis: To address the “temporalization” effect described by Yasir et al. [11], where nerve bundles shift temporally and mask focal damage, segmental analysis was prioritized over global averages. Consequently, RNFL thickness was recorded for the global average (G) and six anatomical sectors: superotemporal (TS), superonasal (NS), nasal (N), inferonasal (NI), inferotemporal (TI), and temporal (T).

Additionally, the automated colour-coded classification generated by the device software was documented based on the normative database: within normal limits (green, *p* > 5%); borderline (yellow, *p* < 5%, outside the 95% normal limits); and abnormal (red, *p* < 1%, outside the 99% normal limits). During the segmentation process, any errors in the automated delineation of the internal limiting membrane (ILM) and RNFL boundaries were manually corrected when necessary. All OCT parameters were calculated using the manufacturer’s integrated analysis software. Despite manual corrections, eyes with persistent inconsistent or suboptimal imaging quality were excluded from the final analysis.

### 2.5. Statistical Analysis

Statistical analyses were performed using IBM SPSS Statistics version 29 (IBM Corp., Armonk, NY, USA). Normality was assessed using the Shapiro–Wilk test. Descriptive data were expressed as mean ± SD and 95% CI. Categorical variables were compared using the chi-square test. Continuous variables among refractive groups were analyzed using one-way ANOVA with Bonferroni correction or the Kruskal–Wallis test with Dunn–Bonferroni post hoc analysis, as appropriate. RNFL measurements across scan diameters (C1–C3) were analyzed using two-way repeated-measures ANOVA to evaluate the effects of refractive group, scan diameter, and their interaction. Sphericity was assessed with Mauchly’s test and corrected using the Greenhouse–Geisser method when necessary. Effect sizes were reported using partial eta squared (ηp^2^) and Cohen’s d. ROC curve analysis was performed to assess diagnostic performance, and Pearson correlation analysis was used to evaluate the relationship between axial length and RNFL thickness. Statistical significance was set at *p* ≤ 0.05. Post hoc power analysis (G*Power v3.1.9.7), based on inferonasal RNFL thickness at the 4.1mm scan diameter, yielded an effect size of d = 0.61 and a statistical power of 0.83.

## 3. Results

### 3.1. Baseline Characteristics

A total of 204 participants were enrolled in the study (66.7% female, 33.3% male): control group (*n* = 41, 20.1%), mild myopia (*n* = 44, 21.6%), moderate myopia (*n* = 66, 32.4%), and high myopia (*n* = 53, 26.0%). The mean age was 26.65 ± 7.73 years, mean BCVA 0.99 ± 0.02, mean IOP 17.04 ± 2.77 mmHg, mean AL 24.90 ± 1.27 mm, mean SE −3.71 ± 2.67 D, and mean central corneal thickness (CCT) 544.51 ± 33.95 µm. No statistically significant differences were observed among groups for age, sex, BCVA, IOP, or CCT (all *p* > 0.05), whereas significant differences were evident for AL and refractive error (both *p* < 0.001) (Table 1).

RNFLT parameters were compared between myopia subgroups and controls at C1, C2, and C3. Analyses were conducted based on quantitative thickness measurements, OCT colour-code distributions, and corresponding ROC curves.

When all cases were evaluated collectively, RNFLT demonstrated a significant progressive reduction across all segments with increasing scan diameter. Mean global RNFLT values by group and scan diameter were: controls 101.1 ± 9.1 µm (C1), 84.8 ± 13.1 µm (C2), 75.3 ± 6.7 µm (C3); mild myopia 98.2 ± 11.8 µm, 83.9 ± 9.5 µm, 73.1 ± 8.0 µm; moderate myopia 101.6 ± 11.2 µm, 85.1 ± 8.0 µm, 74.6 ± 6.1 µm; high myopia 98.7 ± 8.9 µm, 83.5 ± 7.8 µm, 72.4 ± 7.5 µm, respectively (Table 2).

At C1, significant RNFL thinning was observed in the NS (*p* = 0.005) and NI (*p* = 0.037) sectors compared with controls. Posthoc pairwise comparisons (Bonferroni) revealed significant differences in NS between controls and mild myopia (*p* = 0.009) and between moderate and mild myopia (*p* = 0.036). For the NI sector, differences were identified between controls and mild myopia (*p* = 0.056) and between controls and high myopia (*p* = 0.071).

At C2, significant thinning was detected in NS (*p* = 0.008) and NI (*p* = 0.019) sectors. Pairwise comparisons (Bonferroni) demonstrated differences in NS between controls and mild myopia (*p* = 0.048) and between controls and high myopia (*p* = 0.030), and in NI between controls and mild myopia (*p* = 0.051) and between controls and high myopia (*p* = 0.027).

At C3, differences were observed in NS (*p* = 0.019), N (*p* = 0.060), and NI (*p* = 0.049) sectors. However, posthoc comparisons did not reveal statistically significant pairwise differences for NS. Near-significant differences were observed between controls and high myopia in the N sector (*p* = 0.069) and between controls and mild myopia in NI (*p* = 0.066).

### 3.2. RNFL Colour-Code Analysis

Across C1–C3, green colour-code proportions exceeded 80% in all segments. With increasing myopia severity, yellow and red colour-code proportions demonstrated progressive elevation, particularly within the nasal and inferior quadrants.

The highest yellow colour-code proportion occurred in the TI sector at C3 among individuals with high myopia (26.4%; *p* = 0.020). More modest increases were also documented in NI at C1 (*p* = 0.419), NI at C2 (17.0%; *p* = 0.419), and globally at C2 (15.1%; *p*> 0.05). Red colour codes were most prevalent in the N sector at C3 in high myopes (13.2%; *p* = 0.501) and globally at C1 in mild myopes (13.6%; *p*> 0.05).

At C1, the NS sector demonstrated 100% green classification in controls, progressively decreasing with increasing myopia severity (*p* = 0.278). In the NI sector, green classification decreased from 95.1% (controls) to 77.4% (high myopia) with a corresponding increase in yellow classification (*p* = 0.419).

At C2, NS, N, NI, and TI sectors exhibited reduced green and increased yellow/red classifications (e.g., NI: controls 95.1% → high myopia 79.2% green, 17.0% yellow; *p* = 0.419), although differences did not achieve statistical significance (all *p* > 0.05).

At C3, a statistically significant difference was observed exclusively in the TI sector (*p* = 0.020), where green classification decreased from 92.7% (controls) to 71.7% (high myopia), with yellow classification increasing to 26.4%. Differences in other segments did not reach statistical significance (NS: *p* = 0.278; N: *p* = 0.501; NI: *p* = 0.419; T: *p* = 0.152; TS: *p* = 0.544) (Table 3).

### 3.3. Diagnostic Performance (ROC Analysis)

At C1, significant discrimination between myopic and control groups was observed in the NS (AUC = 0.611, *p* = 0.029) and NI (AUC = 0.660, *p* = 0.002) sectors. At C2, both NS (AUC = 0.625, *p* = 0.014) and NI (AUC = 0.674, *p* = 0.001) demonstrated significant discriminatory ability, with NI at C2 exhibiting the highest AUC value overall. At C3, only NI remained statistically significant (AUC = 0.637, *p* = 0.007), while NS (AUC = 0.586, *p* = 0.089), N (AUC = 0.587, *p* = 0.086), and TI (AUC = 0.596, *p* = 0.058) showed borderline significance. No statistically significant differences were detected in other quadrants.

Comparative analysis across scan diameters revealed the strongest discriminative performance at C2, particularly in the NI and NS sectors, followed by C1, whereas C3 exhibited the weakest performance. Across all circular scan diameters, RNFLT in NS and NI sectors was significantly reduced in myopic eyes compared with controls, with this thinning becoming progressively more pronounced with increasing myopia severity. No significant differences were observed in TI, TS, and global measurements. A thinning tendency was noted in the N sector in high myopia, whereas a slight thickening trend was observed in the T sector, neither achieving statistical significance.

**Figure 3:**
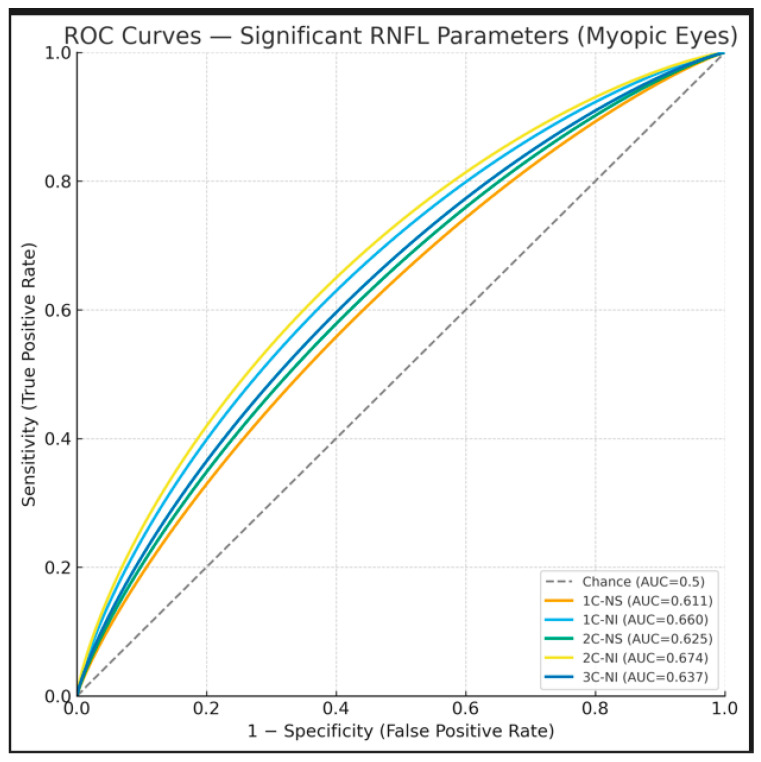
Receiver operating characteristic (ROC) curves of statistically significant RNFL thickness parameters in myopic eyes. Only parameters with *p* < 0.05 are displayed: 3.5-mm scan nasal-superior (C1-NS), 3.5-mm scan nasal-inferior (C1-NI), 4.1-mm scan nasal-superior (C2-NS), 4.1-mm scan nasal-inferior (C2-NI), and 4.7-mm scan nasal-inferior (C3-NI). The diagonal dashed line represents the reference line for chance discrimination (AUC = 0.5).

Overall, NI at C2 achieved the highest discriminative performance (AUC = 0.674, *p* = 0.001), followed by NI at C1 (AUC = 0.660, *p* = 0.002) and C3 (AUC = 0.637, *p* = 0.007) (Table 4, Figure 3). AUC values in other segments were lower and predominantly borderline.

### 3.4. Correlation Between Axial Length and RNFLT

Pearson correlation analysis was performed to evaluate the association between axial length and retinal nerve fibre layer (RNFL) thickness across all scan diameters and sectors (Table 5).

At C1 (3.5 mm), axial length showed significant negative correlations in the nasal (N) sector (r = −0.177, *p* = 0.011) and nasal-inferior (NI) sector (r = −0.264, *p* < 0.001), while a significant positive correlation was observed in the temporal (T) sector (r = 0.208, *p* = 0.003). At C2 (4.1 mm), significant negative correlations were detected in the NI (r = −0.310, *p* < 0.001), N (r = −0.194, *p* = 0.006), and temporal-inferior (TI) (r = −0.231, *p* = 0.001) sectors. The T sector continued to show a significant positive correlation with axial length (r = 0.218, *p* = 0.002). At C3 (4.7 mm), significant negative correlations persisted in the N (r = −0.223, *p* = 0.001), NI (r = −0.261, *p* < 0.001), and TI (r = −0.262, *p* < 0.001) sectors, while the T sector again demonstrated a significant positive correlation (r = 0.198, *p* = 0.004). Global RNFL thickness showed no significant correlation with axial length at C1 or C2 (*p* > 0.05). A weak but statistically significant negative correlation was observed at C3 (r = −0.166, *p* = 0.017).

### 3.5. Repeated-Measures Analysis

**Figure 4:**
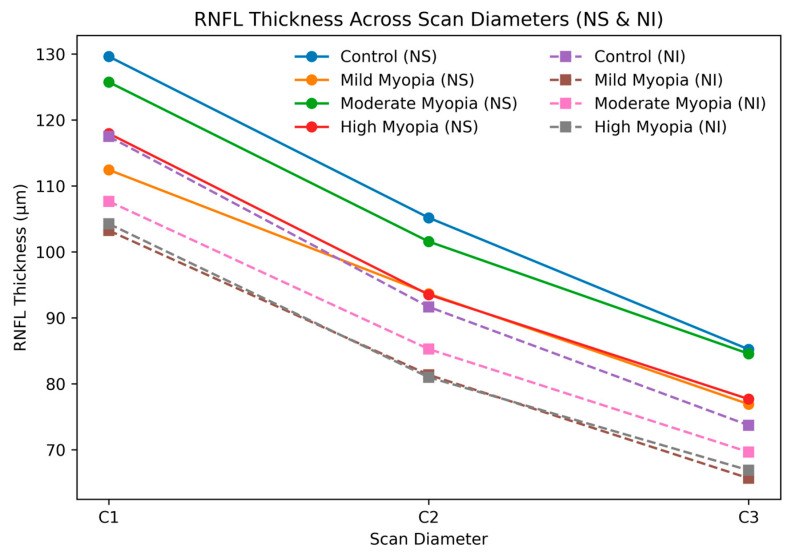
Line plot illustrating mean RNFL thickness across scan diameters (C1–C3) for the nasal-superior (solid lines) and nasal-inferior (dashed lines) sectors. Each colour represents a refractive group (control, mild myopia, moderate myopia, high myopia).

A repeated-measures ANOVA (General Linear Model) was conducted to evaluate RNFLT changes across C1–C3 within and between myopia groups. Mauchly’s test indicated violation of sphericity assumptions for all segments (*p* < 0.05); consequently, Greenhouse–Geisser corrections were applied. Significant between-group differences were primarily observed in nasal segments, including NS (F = 4.240, *p* = 0.006, ηp^2^ = 0.060) and NI (F = 3.186, *p* = 0.025, ηp^2^ = 0.046), indicating small-to-moderate effect sizes and underscoring their sensitivity to myopia-related structural alterations (Table 6, Figure 4). All segments demonstrated highly significant within-subject effects across the three circle diameters (all *p* < 0.001), indicating that RNFLT varied significantly with scan diameter.

Notably, significant group × circle interactions were detected in the NS (F = 3.020, *p* = 0.019) and TS (F = 2.518, *p* = 0.030) sectors, suggesting that the pattern of RNFLT change across diameters differed between myopia groups in these quadrants. No significant interactions were observed in other segments (all *p* > 0.05). These findings demonstrate that RNFLT patterns varied significantly between myopia groups depending on both sector and circle diameter, with nasal quadrants exhibiting the most pronounced differences, corroborating the ROC analysis results.

## 4. Discussion

The diagnosis of glaucoma in myopic eyes represents a substantial clinical challenge. Although RNFL thinning is a primary consequence of glaucoma, similar structural attenuation is frequently observed in myopic eyes due to axial elongation. Consequently, distinguishing between true pathological damage and physiological myopic change is critical. The differentiation between these entities is further complicated by the dual role of myopia: it functions both as an independent risk factor for glaucoma and as a cause of structural and functional changes that mimic glaucomatous pathology [7,28,29]. While OCT enables objective quantification of RNFL thickness, the optimal scanning protocol and circle diameter for evaluating myopic eyes remain subjects of ongoing controversy [7,28,29].

In the present investigation, we addressed this uncertainty by comparing RNFL measurements across three scan diameters (C1–C3) in non-glaucomatous myopic eyes. Significant RNFL thinning was observed across all myopic groups relative to controls. ROC analysis demonstrated the highest discriminative capability in the clinically relevant nasal-inferior (NI) sector at the 4.1 mm (C2) diameter (AUC = 0.674, *p* = 0.001), followed by the nasal-superior (NS) sector (AUC = 0.625, *p* = 0.014). Although overall AUC values indicated moderate discriminatory performance, the 4.1 mm (C2) scan diameter consistently yielded numerically higher AUC values in the clinically relevant NS and NI sectors compared with both the standard 3.5 mm (C1) and larger 4.7 mm (C3) diameters (e.g., NI sector AUC: 0.674 for C2 vs. 0.660 for C1 and 0.637 for C3).

This consistent pattern suggests that the 4.1 mm diameter offers a superior balance between sensitivity and specificity. By shifting the scan circle slightly away from the immediate peripapillary region, C2 reduces susceptibility to myopia-related structural confounders such as peripapillary atrophy and disc tilt—factors that can contribute to false-positive classifications in standard 3.5 mm scans. Simultaneously, it remains sufficiently close to the optic disc margin to preserve adequate sensitivity for detecting structural alterations, which may become attenuated at larger diameters (C3) due to physiological RNFL thinning. Taken together, these findings suggest that the 4.1 mm protocol represents a potentially advantageous intermediate scanning diameter that may help reduce diagnostic ambiguity.

To understand the pathophysiology behind this specific distribution, one must consider the biomechanics of axial elongation. Axial elongation produces generalized RNFL thinning through redistribution of axons over an expanded retinal surface [10,23,28,29]. However, this thinning is not uniformly distributed. In our cohort, thinning was most pronounced in the NS and NI sectors, whereas temporal quadrants demonstrated relative preservation. While Mishra et al. reported findings supporting a “nasalization” model [10], our results demonstrate greater concordance with the “temporalization” (temporal shifting) hypothesis described by Savini et al. [23], Leung et al. [30], and Wagner et al. [31]. According to this model, axial elongation results in temporal displacement of the retina, inducing stretching and thinning of nasal fibres while producing compression of temporal fibres. Wagner et al. demonstrated that each 1 mm increase in AL reduced the superior–inferior RNFL peak-angle difference by 5.9° [31]. The distinct nasal thinning observed in our study aligns with this mechanism of temporal retinal displacement.

From a clinical perspective, this pattern contrasts markedly with the classical glaucomatous pattern, wherein early structural loss predominantly affects the TI and TS bundles [32,33,34,35]. Conversely, our demonstration of predominant nasal thinning indicates that these two conditions leave distinctly opposite anatomical signatures on the ONH. This distinction is critical because standard OCT normative databases, which predominantly comprise non-myopic eyes, frequently misinterpret this physiological nasal thinning as pathological. Such misclassification underlies the “red disease” phenomenon [12].

Beyond anatomical distribution, the accuracy of these measurements is fundamentally influenced by ocular optics. Axial length is a fundamental determinant of the actual physical size of circumpapillary retinal scans [28]. According to the ocular magnification model (Littmann–Bennett), an increase in axial length causes a constant angular scan to subtend a larger linear distance on the retinal surface [36,37]. Our correlation analysis highlights that the choice of scan diameter is a critical determinant of how this bias manifests. While the C3 scan showed a significant negative correlation with AL in global thickness (r = −0.166, *p* = 0.017), the C2 diameter maintained a non-significant relationship (r = −0.074, *p* = 0.292). This suggests that the 4.1 mm diameter functions as a functional “neutral zone” where opposing sources of axial length-related bias—namely magnification-induced sampling shifts and actual anatomical fibre distribution—effectively reach a compensatory equilibrium.

In addition to optical factors, the choice of centering method for circumpapillary RNFL measurements substantially influences measurement accuracy, particularly in myopic eyes with optic disc tilt and peripapillary remodelling. Conventional optic disc–centred scans, typically acquired at a fixed diameter of approximately 3.4–3.5 mm, may be more susceptible to anatomical distortion caused by disc torsion, parapapillary atrophy, and asymmetric scleral stretching. These factors can alter scan alignment and contribute to artifactual RNFL thinning and increased false-positive classifications [38,39] In contrast, Bruch’s membrane opening (BMO)–centred approaches use a more anatomically consistent reference landmark and have been reported to improve scan localization and measurement reproducibility in eyes with tilted or myopic optic discs. Sawada et al. demonstrated that BMO-centred measurements provide more accurate scan positioning compared with conventional disc-centred methods, thereby reducing alignment-related errors [26]. Similarly, Lee et al. reported superior diagnostic performance of BMO-centred RNFL assessment in tilted discs, with significantly higher AUC values compared with disc-centred analysis (AUC: 0.933 vs. 0.843, *p* = 0.027) [27]. Moreover, Rebolleda et al. showed that BMO–minimum rim width (BMO-MRW) analysis yielded substantially lower false-positive rates than conventional RNFL thickness measurements in eyes with disc tilt (8% vs. 62%, *p* < 0.001) [40]. Collectively, these data suggest that BMO-centred imaging provides a more anatomically stable framework for peripapillary assessment in structurally complex optic discs. The use of BMO-centred concentric scan diameters in the present study is therefore aligned with prior methodological recommendations and may have contributed to the measurement stability observed, particularly at the intermediate (C2) scan diameter.

The clinical utility of this optimized protocol is further substantiated by the analysis of 95% confidence intervals (CIs). Beyond statistical significance (*p*-values), the evaluation of CIs offers additional insight into diagnostic precision. Notably, in the nasal-inferior (NI) sector at the 4.1 mm diameter, the 95% CIs of the control and high myopia groups did not overlap. Specifically, the lower bound of the control group (86.87 µm) remained slightly higher than the upper bound of the high myopia group (86.21 µm). This non-overlapping pattern suggests that the detected thinning is unlikely to represent a trivial numerical fluctuation and instead reflects a measurable structural shift. Accordingly, the 4.1 mm protocol minimizes diagnostic ambiguity, offering a robust tool to distinguish healthy myopic eyes from those with potential pathology.

## 5. Study Limitations

This study has several limitations. Although the data were collected prospectively, the cross-sectional design precludes assessment of longitudinal structural change; therefore, the temporal stability and predictive significance of the observed nasal thinning cannot be established. The single-centre setting and relatively homogeneous study population may limit generalizability. In addition, exclusion of glaucoma suspects and early glaucoma cases restricts evaluation of diagnostic thresholds in clinically equivocal scenarios. Potential confounding variables, including axial length–related magnification effects, optic disc morphology, and segmentation variability in highly myopic eyes, may have influenced RNFL measurements despite careful quality control. Furthermore, the absence of normative databases calibrated for non-standard scan diameters (4.1 mm and 4.7 mm) may affect classification accuracy. Accordingly, our findings should be interpreted as exploratory and hypothesis-generating rather than definitive evidence of diagnostic superiority. Multicentre and longitudinal validation studies are warranted to confirm the clinical utility of intermediate scan diameters in myopic populations.

## 6. Conclusions

RNFL thinning in non-glaucomatous myopic eyes was predominantly localized to the nasal-superior and nasal-inferior sectors, demonstrating a distribution pattern distinct from the temporal-predominant loss typically observed in glaucoma. Among the evaluated scan diameters, the 4.1 mm (C2) protocol showed the highest discriminatory performance in the NI sector and demonstrated relatively greater stability with respect to axial length–related variation. These findings suggest that intermediate scan diameters may provide a more balanced structural assessment in myopic eyes. However, given the cross-sectional design and methodological constraints, the present results should be interpreted as exploratory and hypothesis-generating. Further longitudinal and multicentre studies are required to validate the clinical applicability and diagnostic performance of the 4.1 mm scan diameter in diverse myopic populations.

## Figures and Tables

**Table 1:** Demographic and clinical characteristics of the study groups.

Parameter (Mean ± SD)	Control (*n* = 41)	Mild Myopia (*n* = 44)	Moderate Myopia (*n* = 66)	High Myopia (*n* = 53)	Total (*n* = 204)	*p*-Value
Age (years)	26.63 ± 6.29	26.02 ± 7.17	26.73 ± 8.10	27.08 ± 8.83	26.65 ± 7.73	0.695
Gender	26 (63.4)/	26 (59.1)/	48 (72.7)/	36 (67.9)/	136 (66.7)/	0.483
(F/M), n (%)	15 (36.6)	18 (40.9)	18 (27.3)	17 (32.1)	68 (33.3)	
Visual acuity (decimal)	1.00 ± 0.00	1.00 ± 0.00	0.99 ± 0.02	0.99 ± 0.03	0.99 ± 0.02	0.365
Intraocular pressure (mmHg)	16.49 ± 2.24	17.14 ± 2.97	17.35 ± 2.64	17.00 ± 3.11	17.04 ± 2.77	0.211
Degree of myopia (D)	−0.23 ± 0.32	−1.87 ± 0.50	−4.27 ± 0.91	−7.23 ± 1.10	−3.71 ± 2.67	<0.001 *
Central corneal thickness (µm)	545.32 ± 36.86	538.23 ± 25.76	545.52 ± 33.65	547.87 ± 38.01	544.51± 33.95	0.554
Axial length (mm)	23.62 ± 0.68	24.17 ± 0.78	25.10 ± 0.86	26.23 ± 0.96	24.90 ± 1.27	<0.001 *

D—diopters; F—female; M—male; SD—standard deviation; µm—micrometer. The forward slash (/) indicates female/male distribution. Mean ± SD for continuous variables and n (%) for categorical variables. * Indicates statistical significance at *p* < 0.05.

**Table 2:** Comparison of global and sectoral circumpapillary RNFL thickness among refractive error groups at three scan diameters (3.5 mm, 4.1 mm, and 4.7 mm).

Scan Diameter	Segment	Control Mean ± SD (95% CI)	Mild Myopia Mean ± SD (95% CI)	Moderate Myopia Mean ± SD (95% CI)	High Myopia Mean ± SD (95% CI)	*p*-Value
C1	G	101.15 ± 9.13 98.26–104.03	98.16 ± 11.80 94.57–101.75	101.65 ± 11.27 98.88–104.42	98.66 ± 8.93 96.20–101.12	0.225
C1	NS	129.61 ± 19.86 123.34–135.88	112.43 ± 29.06 103.59–121.27	125.73 ± 27.85 118.88–132.57	117.94 ± 18.95 112.72–123.17	0.005 **
C1	N	81.07 ± 11.48 77.45–84.70	79.25 ± 13.21 75.23–83.27	79.97 ± 16.40 75.94–84.00	77.74 ± 18.39 72.66–82.81	0.757
C1	NI	117.49 ± 21.88 110.58–124.3	103.23 ± 27.66 94.82–111.64	107.65 ± 22.64 102.08–113.22	104.25 ± 27.74 96.60–111.89	0.037 *
C1	TI	151.49 ± 19.75 145.25–157.72	151.98 ± 19.72 145.98–157.97	160.88 ± 79.45 141.35–180.41	145.40 ± 21.64 139.43–151.36	0.375
C1	T	71.85 ± 10.22 68.63–75.08	73.02 ± 9.13 70.25–75.80	75.65 ± 10.41 73.09–78.21	75.58 ± 14.75 71.52–79.65	0.263
C1	TS	127.85 ± 21.84 120.96–134.75	132.07 ± 19.50 126.14–138.00	133.41 ± 20.29 128.42–138.40	133.25 ± 22.48 127.05–139.44	0.558
C2	G	84.78 ± 13.15 80.63–88.93	83.91 ± 9.51 81.02–86.80	85.15 ± 7.97 83.19–87.11	83.45 ± 7.77 81.31–85.60	0.773
C2	NS	105.17 ± 18.19 99.43–110.91	93.66 ± 19.69 87.67–99.65	101.56 ± 23.83 95.70–107.42	93.51 ± 14.86 89.41–97.61	0.008 **
C2	N	67.05 ± 8.95 64.22–69.88	65.61 ± 10.14 62.53–68.70	65.91 ± 11.03 63.20–68.61	63.77 ± 13.79 59.97–67.58	0.551
C2	NI	91.66 ± 15.16 86.87–96.45	81.36 ± 19.45 75.45–87.28	85.26 ± 17.31 81.00–89.51	80.98 ± 18.96 75.75–86.21	0.019 *
C2	TI	134.93 ± 17.44 129.42–140.43	133.09 ± 18.22 127.55–138.63	133.67 ± 12.61 130.57–136.77	128.98 ± 16.32 124.48–133.48	0.270
C2	T	63.61 ± 8.82 60.82–66.40	65.52 ± 7.60 63.21–67.83	67.20 ± 9.01 64.98–69.41	67.55 ± 12.36 64.14–70.96	0.186
C2	TS	117.51 ± 19.74 111.28–123.75	119.86 ± 16.83 114.75–124.98	119.11 ± 17.23 114.87–123.34	120.75 ± 18.25 115.72–125.79	0.849
C3	G	75.29 ± 6.66 73.19–77.39	73.11 ± 8.03 70.67–75.55	74.55 ± 6.05 73.06–76.03	72.36 ± 7.48 70.30–74.42	0.158
C3	NS	85.22 ± 14.65 80.59–89.85	76.91 ± 16.50 71.89–81.93	84.56 ± 20.09 79.62–89.50	77.68 ± 13.63 73.92–81.44	0.019 *
C3	N	57.15 ± 7.37 54.82–59.47	55.52 ± 8.56 52.92–58.13	55.61 ± 9.31 53.32–57.90	51.79 ± 13.46 48.08–55.50	0.060
C3	NI	73.71 ± 13.06 69.58–77.83	65.68 ± 15.25 61.04–70.32	69.65 ± 14.01 66.21–73.10	66.89 ± 15.08 62.73–71.04	0.049 *
C3	TI	122.15 ± 14.65 117.52–126.77	118.48 ± 16.37 113.50–123.46	117.82 ± 11.88 114.90–120.74	115.06 ± 13.75 111.26–118.85	0.116
C3	T	58.15 ± 8.30 55.53–60.77	59.98 ± 6.48 58.01–61.95	61.32 ± 8.60 59.20–63.43	61.83 ± 10.44 58.95–64.71	0.175
C3	TS	109.46 ± 16.63 104.21–114.71	107.55 ± 13.88 103.32–111.77	106.73 ± 19.60 101.91–111.55	110.57 ± 14.32 106.62–114.52	0.603

C1—circumpapillary scan at 3.5 mm; C2—circumpapillary scan at 4.1 mm; C3—circumpapillary scan at 4.7 mm; RNFL—retinal nerve fibre layer thickness; G—global; NS—superonasal; N—nasal; NI—inferonasal; TI—inferotemporal; T—temporal; TS—superotemporal; SD—standard deviation; CI—confidence interval. Data are presented as mean ± SD with 95% confidence intervals (CI). p-values indicate overall group differences obtained by one-way analysis of variance (ANOVA). * *p* < 0.05; ** *p* < 0.01.

**Table 3:** Distribution of RNFL colour codes (%) across myopia groups at the 3rd circle scan diameter.

Segment	Group	Green *n* (%)	Yellow *n* (%)	Red *n* (%)	χ^2^	*p*-Value
NS	Control	41 (100)	0 (0)	0 (0)	7.485	0.278
	Mild Myopia	41 (93.2)	3 (6.8)	0 (0)		
	Moderate Myopia	61 (92.4)	3 (4.5)	2 (3.0)		
	High Myopia	49 (92.5)	4 (7.5)	0 (0)		
N	Control	38 (92.7)	1 (2.4)	2 (4.9)	5.344	0.501
	Mild Myopia	38 (86.4)	4 (9.1)	2 (4.5)		
	Moderate Myopia	55 (83.3)	6 (9.1)	5 (7.6)		
	High Myopia	42 (79.2)	4 (7.5)	7 (13.2)		
NI	Control	39 (95.1)	2 (4.9)	0 (0)	6.035	0.419
	Mild Myopia	35 (79.5)	8 (18.2)	1 (2.3)		
	Moderate Myopia	56 (84.8)	9 (13.6)	1 (1.5)		
	High Myopia	46 (86.8)	5 (9.4)	2 (3.8)		
TI	Control	38 (92.7)	2 (4.9)	1 (2.4)	15.061	0.020 *
	Mild Myopia	35 (79.5)	7 (15.9)	2 (4.5)		
	Moderate Myopia	58 (87.9)	4 (6.1)	4 (6.1)		
	High Myopia	38 (71.7)	14 (26.4)	1 (1.9)		
T	Control	38 (92.7)	3 (7.3)	0 (0)	9.404	0.152
	Mild Myopia	44 (100)	0 (0)	0 (0)		
	Moderate Myopia	64 (97.0)	2 (3.0)	0 (0)		
	High Myopia	52 (98.1)	0 (0)	1 (1.9)		
TS	Control	39 (95.1)	0 (0)	2 (4.9)	5.001	0.544
	Mild Myopia	40 (90.9)	2 (4.5)	2 (4.5)		
	Moderate Myopia	60 (90.9)	5 (7.6)	1 (1.5)		
	High Myopia	50 (94.3)	2 (3.8)	1 (1.9)		

NS—nasal-superior; N—nasal; NI—nasal-inferior; T—temporal; TS—temporal-superior; TI—temporal-inferior. * Statistically significant (*p* < 0.05).

**Table 4:** Area under the ROC curves (AUC) of RNFL thickness parameters in circle scans with different diameters in myopic eyes.

Variables (Segment)	AUC	95% CI (LB–UB)	*p*-Value
C1–G	0.541	0.444–0.639	0.412
C1–NS	0.611	0.520–0.701	0.029 *
C1–N	0.542	0.450–0.634	0.407
C1–NI	0.660	0.575–0.744	0.002 **
C1–TI	0.528	0.427–0.629	0.584
C1–T	0.429	0.327–0.530	0.158
C1–TS	0.444	0.344–0.545	0.270
C2–G	0.548	0.447–0.648	0.345
C2–NS	0.625	0.533–0.717	0.014 *
C2–N	0.569	0.479–0.660	0.170
C2–NI	0.674	0.592–0.756	0.001 **
C2–TI	0.557	0.457–0.657	0.261
C2–T	0.413	0.311–0.514	0.084 †
C2–TS	0.475	0.371–0.579	0.617
C3–G	0.561	0.464–0.658	0.230
C3–NS	0.586	0.491–0.681	0.089 †
C3–N	0.587	0.497–0.677	0.086 †
C3–NI	0.637	0.552–0.722	0.007 **
C3–TI	0.596	0.501–0.690	0.058 †
C3–T	0.407	0.304–0.509	0.065 †
C3–TS	0.532	0.432–0.633	0.521

AUC—area under the curve; CI—confidence interval; LB—lower bound; UB—upper bound; C1, C2, C3—circular scans at 3.5, 4.1, and 4.7 mm diameters; G—global; NS—nasal-superior; N—nasal; NI—nasal-inferior; TI—temporal-inferior; T—temporal; TS—temporal-superior. † *p* < 0.10; * *p* < 0.05; ** *p* < 0.01.

**Table 5:** Segment-based correlations between axial length and RNFL thickness across scan diameters.

Segment	C1 (3.5 mm)		C2 (4.1 mm)		C3 (4.7 mm)	
	r	*p*	r	*p*	r	*p*
G	−0.070	0.314	−0.074	0.292	−0.166	0.017 *
NS	−0.068	0.328	−0.079	0.261	−0.053	0.448
N	−0.177	0.011 *	−0.194	0.005 **	−0.223	0.001 **
NI	−0.264	<0.001 **	−0.310	<0.001 **	−0.261	<0.001 **
TI	−0.126	0.071	−0.231	0.001 **	−0.262	<0.001 **
T	+0.208	0.003 **	+0.218	0.002 **	+0.198	0.004 **
TS	+0.074	0.288	+0.058	0.411	+0.046	0.508

* *p* < 0.05; ** *p* < 0.01; G—global; NS—nasal-superior; N—nasal; NI—nasal-inferior; TI—temporal-inferior; T—temporal; TS—temporal-superior.

**Table 6:** Repeated-measures ANOVA results for RNFL thickness across three circle scan diameters (C1: 3.5 mm, C2: 4.1 mm, C3: 4.7 mm) in different segments.

Segment	Group Effect (F, *p*)	Circle Scan Effect (F, *p*)	Group × Circle Scan (F, *p*)	Significance
G	1.158, 0.327	1671.295, <0.001 ***	0.953, 0.453	Circle scan only
NS	4.240, 0.006 **	1359.716, <0.001 ***	3.020, 0.019 *	All three significant
N	1.023, 0.383	845.605, <0.001 ***	0.465, 0.783	Circle scan only
NI	3.186, 0.025 *	1079.162, <0.001 ***	1.556, 0.194	Group & Circle scan
TI	1.303, 0.275	84.880, <0.001 ***	0.958, 0.415	Circle scan only
T	1.553, 0.202	1358.690, <0.001 ***	0.603, 0.658	Circle scan only
TS	0.272, 0.845	422.104, <0.001 ***	2.518, 0.030 *	Circle scan & Interaction

* *p* < 0.05, ** *p* < 0.01, *** *p* < 0.001; G—global; NS—nasal-superior; N—nasal; NI—nasal-inferior; TI—temporal-inferior; T—temporal; TS—temporal-superior.

## Data Availability

The datasets generated and/or analyzed during the current study are available from the corresponding author on reasonable request.

## Abbreviations

AL	Axial length
ANOVA	Analysis of variance
ART	Automatic real-time tracking
AUC	Area under the ROC curve
BCVA	Best-corrected visual acuity
BMO	Bruch’s membrane opening
C1/C2/C3	Circumpapillary circle scans at 3.5/4.1/4.7 mm
CCT	Central corneal thickness
G	Global RNFL thickness (device output)
GMPE	Glaucoma Module Premium Edition
ILM	Internal limiting membrane
IOP	Intraocular pressure
N	Nasal quadrant
NI	Nasal-inferior quadrant
NS	Nasal-superior quadrant
OCT	Optical coherence tomography
OHT	Ocular hypertension
ONH	Optic nerve head
PPA	Parapapillary atrophy
POAG	Primary open-angle glaucoma
RNFL	Retinal nerve fibre layer
RNFLT	Retinal nerve fibre layer thickness
ROC	Receiver operating characteristic
SD-OCT	Spectral-domain optical coherence tomography
SE	Spherical equivalent refraction
SLD	Superluminescent diode
T	Temporal quadrant
TI	Temporal-inferior quadrant
TS	Temporal-superior quadrant
